# Rapid Screening Alpha-Glucosidase Inhibitors from Polygoni Vivipari Rhizoma by Multi-Step Matrix Solid-Phase Dispersion, Ultrafiltration and HPLC

**DOI:** 10.3390/molecules26206111

**Published:** 2021-10-10

**Authors:** Haoxiang Li, Zhuobin He, Qianhui Shen, Weifeng Fan, Guoying Tan, Yuansheng Zou, Quanxi Mei, Zhengming Qian

**Affiliations:** 1 Studio of National Chinese Medical Science Master Shiyuan Jin, Bao’an Authentic TCM Therapy Hospital, Shenzhen 518101, China; lihaoxiang199411@163.com; 2 Key Laboratory of State Administration of Traditional Chinese Medicine, Dongguan HEC Cordyceps R&D Co., Ltd., Dongguan 523850, China; hezhuobin@HEC.CN (Z.H.); Caroline_qianhui@163.com (Q.S.); 20193121214@stu.gzucm.edu.cn (W.F.); tanguoying@HEC.CN (G.T.); zouyuansheng@HEC.CN (Y.Z.)

**Keywords:** Polygoni Vivipari Rhizoma, alpha-glucosidase inhibitors, MSPD, ultrafiltration, HPLC

## Abstract

Polygoni Vivipari Rhizoma (PVR), the dried root of *Polygonum viviparum*, has been used as herbal medicine in China for a long time. In the present study, a new method based on multi-step matrix solid-phase dispersion (MSPD), ultrafiltration and high performance liquid chromatography (HPLC) for screening alpha-glucosidase inhibitors (AGIs) from PVR was proposed. First, three different PVR extractions were prepared by multi-step MSPD with 15% methanol, 60% methanol and 100% methanol. Second, the alpha-glucosidase inhibition tests for the three extracts were carried out, and the 60% methanol extraction showed the best activity. Then, the AGIs screening experiment was performed with ultrafiltration and HPLC analysis using the 60% methanol extraction. Seven binding components (quercetin−3−*O*−vicianoside, quercetin 3−*O*−neohesperidoside, rutin, hyperoside, quercetin 3−*O*−glucuronide, luteolin−7−*O*−neohesperidoside, kaempferol 3−glucuronide) were found. These seven components were further validated as the AGIs by molecular docking analysis. The developed method was a rapid and efficient tool for screening AGIs from PVR, which provided scientific data for the bioactive components study of PVR.

## 1. Introduction

Diabetes is one of the most serious chronic diseases, resulting from relative insulin deficiency [1]. There are more than 400 million people in the world that have been diagnosed with type 2 diabetes [2]. Alpha-glucosidase inhibitors (AGIs), one of the first-line therapeutic drugs that can delay the absorption of carbohydrates and regulate postprandial blood glucose, are widely used as hypoglycemics to treat type 2 diabetes [3,4]. However, adverse effects of some synthetic AGIs have been found in clinical trials [5]. Therefore, obtaining safe and efficient AGIs is of significant importance for the treatment of diabetes. Herbal medicines are a rich source of natural, active components with few harmful side effects, which have been used for thousands of years. Therefore, screening natural AGIs from herbal medicine is a good strategy. However, herbal medicine is a complex mixture containing various compounds. The traditional method for screening of active compounds from herbal medicines, including sample extraction, separation, purification and activity testing, is laborious and time-consuming. Ultrafiltration combined with high performance liquid chromatography (HPLC) was proved as a powerful approach for discovering potential bioactive compounds from complex mixtures, which has been successfully applied in screening of alpha-glucosidase inhibitors, tyrosinase inhibitors and xanthine oxidase inhibitors from herbal medicines [6,7,8]. However, the above mentioned methods used the common sample preparation, such as ultrasonic extraction and reflux extraction. It is difficult to simultaneously extract hydrophilic and hydrophobic components by one solvent. Fortunately, the multi-step matrix solid phase dispersion (MSPD) can extract different polarity components from herbal medicine by multi-step elution with different solvents [9,10,11]. Thus, development of a method combining multi-step MSPD, ultrafiltration and HPLC would be a reasonable strategy for fast and comprehensive screening of active components from herbal medicines.

Polygoni Vivipari Rhizoma (PVR), which is derived from the dried root of *Polygonum viviparum*, is a traditionally used herbal medicine and known as “Ranbu” in Chinese. It has been used in stopping diarrhea and activating blood circulation to dissipate blood stasis [12]. Modern studies have shown that PVR contains many components, such as flavonoids, organic acids, sterols, polysaccharides, and so on [13,14]. Our preliminary test indicated that PVR possesses good anti-oxidant and alpha-glucosidase inhibition activity. The anti-oxidant components from PVR have been reported and eighteen bioactive components were found [15]. The hypoglycemic components in PVR are still unknown. Therefore, in the current study, a new method based on multi-step MSPD, ultrafiltration and HPLC analysis, for the screening of active components from herbal medicines was developed. The developed method was successfully applied in analysis of AGIs from PVR, and seven AGIs were found. In addition, the binding sites and interactions between AGIs and alpha-glucosidase were analyzed by molecular docking.

## 2. Results and Discussion

### 2.1. Optimization of Multi-Step MSPD Extraction Conditions

The PVR sample contains various hydrophilic and hydrophobic compounds. With traditional extraction methods, it is difficult to get the different polar components by use of a single solvent. Thus, the multi-step MSPD method was selected to extract the components from PVR by different solvents. In order to obtain the optimum multi-step MSPD method, different extraction conditions (the ratio of sample-dispersant, the polarity and volume of eluting solvent) were studied by the single-factor method. According to previous reports, methanol was commonly used as the extract solvent because it is practical and has superior capacity for extracting components from herbal medicine [10,15]. Diatomaceous earth, which has been used in component extraction of PVR, was chosen as the dispersant based on its excellent dispersion capacity and low interference [16].

First, three different ratios of sample–dispersant (1:1, 1:2, 1:4) were compared. The results showed that the sample–dispersant ratio of 1:1 had low extraction efficiency, while the ratios of 1:2 and 1:4 had similar extraction efficiency. Therefore, the sample–dispersant ratio of 1:2 was used for sample extraction to save materials. Second, the elution methanol of different concentrations could elute the different target components according to their similar polarity. To obtain different polar fractions of PVR, the concentration of elution solutions was studied in two consecutive steps (10%/15%/20% methanol in step one; 60%/65%/70% methanol in step two). The results of step one showed that 10% methanol solution required more volume and time for eluting the polar compounds than the other two concentrations (15%/20%). Further, the extraction efficiency of 15% methanol and 20% methanol were similar. Thus, the 15% methanol was chosen to elute the polar component fraction based on lower organic solvent cost. The middle polarity fraction could be rapidly eluted by increasing the methanol concentration to 60%/65%/70%. Based on the lower methanol cost, 60% methanol was selected to extract the middle polar components. At the end, 100% methanol was used to elute the weakly polar compounds. The influence of different elution solvent volumes (10 mL, 20 mL, 30 mL, 40 mL, and 50 mL) was also tested. Testing revealed that 40 mL was sufficient to extract the components for three methanol concentrations (15%, 60% and 100%). Therefore, the elution solution volume was fixed at 40 mL. In summary, the optimal condition of multi-step MSPD extraction was as follows; the ratio of sample-dispersant was 1:2, and 40 mL of 15%, 60%, 100% methanol was used to elute different polar component fractions from PVR samples, respectively.

### 2.2. The Optimization of HPLC Conditions

Poroshell column, which has the advantages of high resolution and low pressure, has been widely applied in rapid HPLC separation of herbal medicine samples [11,15]. In the current experiment, a Poroshell 120 SB-Aq column was used for PVR sample separation. Three different mobile phases, including 0.2% acetic acid-acetonitrile, 0.1% formic acid-acetonitrile, and water-acetonitrile, were tested. The results showed that 0.2% acetic acid-acetonitrile as the mobile phase presented satisfying chromatographic peaks and high separation efficiency. In addition, different detected wavelengths (254 nm, 330 nm and 360 nm) were compared for the detection of compounds from PVR [17]. The results showed that higher peak responses and smoother baselines were obtained at 360 nm.

### 2.3. Alpha-Glucosidase Inhibitory Activity of Different PVR Fractions

**Figure 1:**
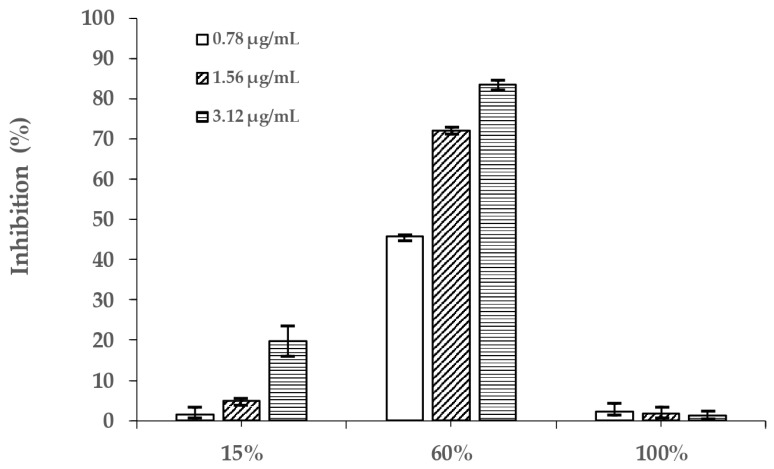
The inhibition (%) of three PVR fractions.

The alpha-glucosidase inhibitory activity of three different PVR fractions (0.78, 1.56, 3.12 µg/mL) are presented in Figure 1. The 60% methanol PVR fraction had the best alpha-glucosidase inhibition (45.80 ± 0.09, 72.09 ± 0.76, and 83.34 ± 1.14%), followed by the 15% methanol PVR fraction (1.61 ± 1.61, 4.97 ± 0.78, and 19.81 ± 3.68%) and the 100% methanol PVR fraction (2.14 ± 2.13, 1.75 ± 1.74, and 1.28 ± 1.15%). These results indicate that the 60% methanol PVR fraction may contain the highest amount of AGIs. Consequently, the 60% methanol PVR fraction was selected for further ultrafiltration and HPLC experiments.

### 2.4. Identification of Components from PVR

**Figure 2:**
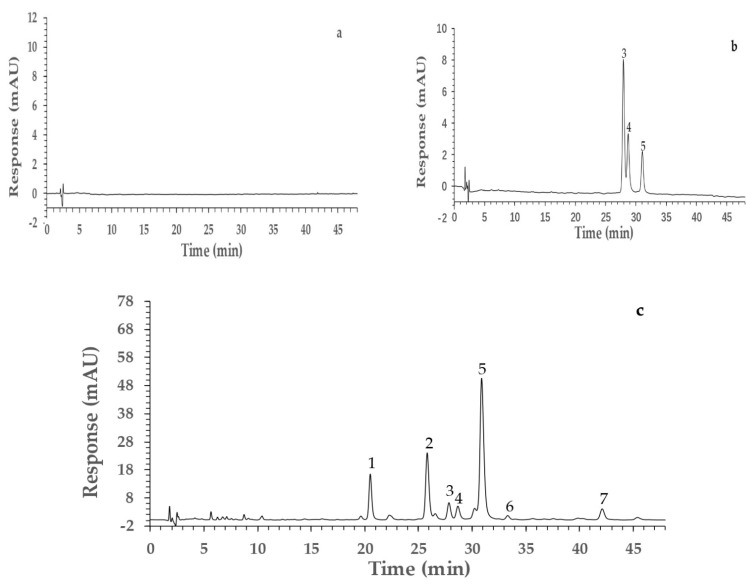
The HPLC chromatograms of a blank sample (**a**), reference compounds (**b**), and PVR (**c**). Quercetin−3−O−vicianoside (**1**), quercetin 3−*O*−neohesperidoside (**2**), rutin (**3**), hyperoside (**4**), quercetin−3−*O*−glucuronide (**5**), luteolin−7−*O*−neohesperidoside (**6**), kaempferol 3−glucuronide (**7**).

**Figure 3:**
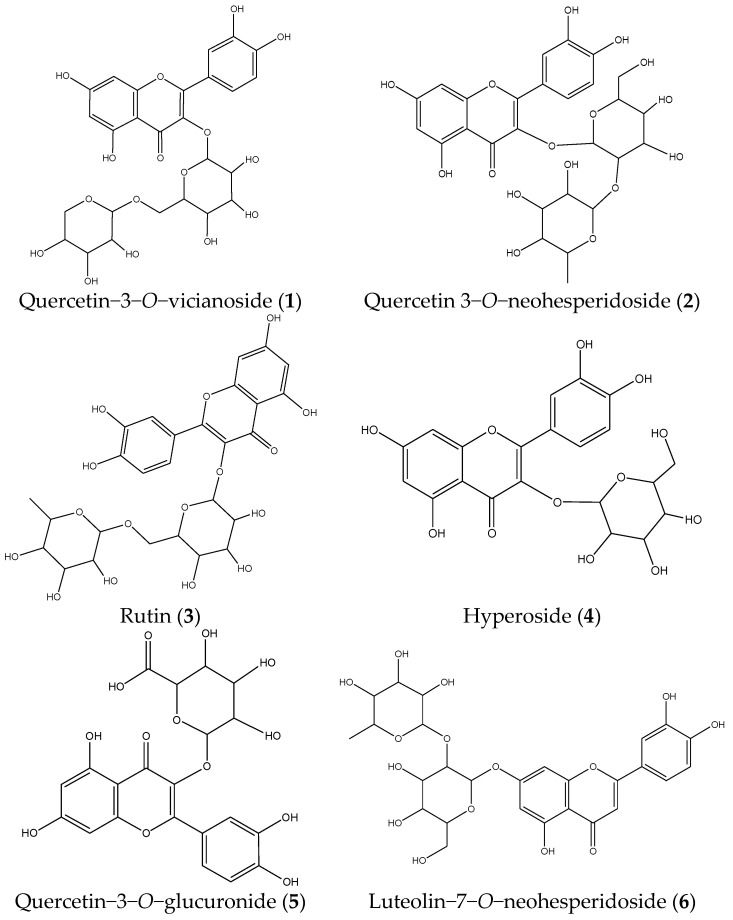

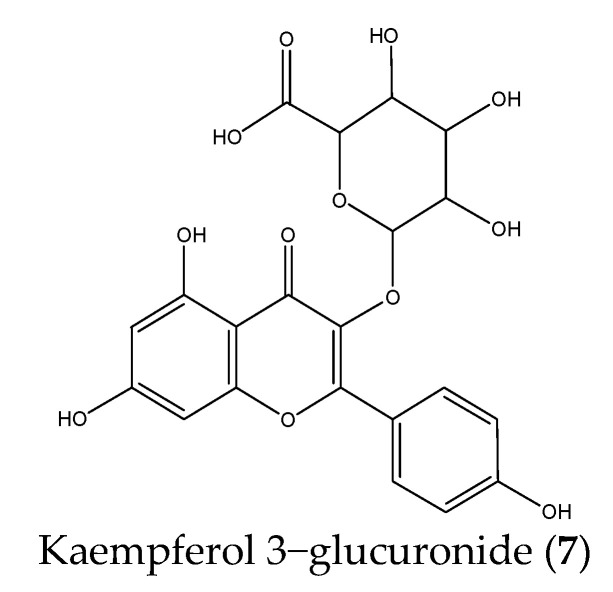
The chemical structures of the seven compounds.

The 60% methanol fraction of PVR exhibited excellent alpha-glucosidase inhibitory activity, but the chemical compounds were unclear. Therefore, the HPLC-UV-MS experiment was carried out to identify the components from it. The HPLC chromatograms are presented in Figure 2 and the MS identification results are summarized in Table 1. The specificity test showed that there were no interfering peaks in the blank sample chromatogram (Figure 2a) and the chromatographic peaks of the reference compounds were found in the PVR chromatogram (Figure 2b,c). Three chromatographic peaks (peaks **3–5**) were identified as rutin (**3**), hyperoside (**4**), and quercetin−3−*O*−glucuronide (**5**) by comparing MS data and retention time with reference compounds. The other four chromatographic peaks were identified as quercetin−3−*O*−vicianoside (**1**), quercetin 3−*O*−neohesperidoside (**2**), luteolin−7−*O*−neohesperidoside (**6**), and kaempferol 3−glucuronide (**7**), by comparing the MS data with the MassBank database and literature [18,19,20,21]. The chemical structures of these compounds are shown in Figure 3.

### 2.5. Screening of Potential AGI by Ultrafiltration and HPLC Analysis

**Figure 4:**
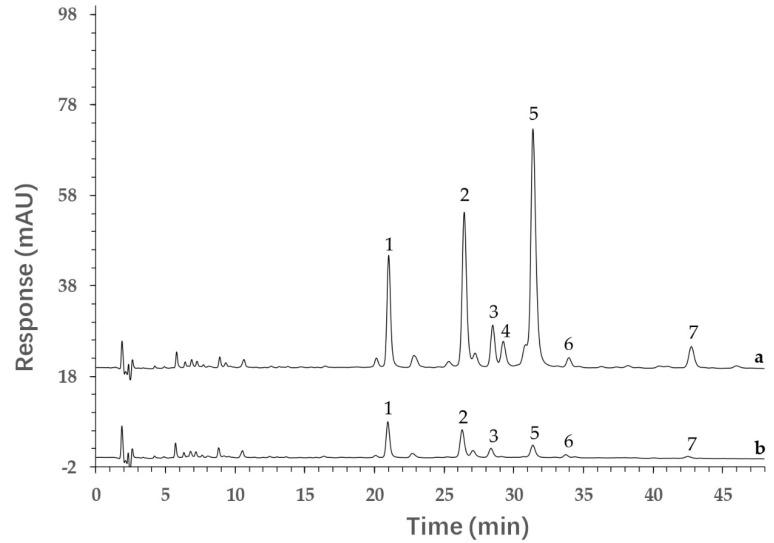
The chromatograms of PVR interacted with alpha-glucosidase. (**a**) Control group (with inactive alpha-glucosidase); (**b**) experimental group (with active alpha-glucosidase). Quercetin−3−*O*−vicianoside (**1**), quercetin 3−*O*−neohesperidoside (**2**), rutin (**3**), hyperoside (**4**), quercetin−3−*O*−glucuronide (**5**), luteolin−7−*O*−neohesperidoside (**6**), kaempferol 3−glucuronide (**7**).

The ultrafiltration combined with HPLC analysis method was employed for screening the potential AGIs in the 60% methanol PVR fraction. The ultrafiltrates were analyzed by HPLC, and then the potential bioactive components were found by comparing the peak areas of detected compounds in the control group (incubated with inactive alpha-glucosidase) and experimental group (incubated with active alpha-glucosidase). The potential bioactive components interacted with active enzymes and the content in ultrafiltrates was reduced. The peak areas of potential AGIs in the experimental group were obviously lower than that in the control group. Figure 4 shows the ultrafiltration and HPLC results of the 60% methanol PVR fraction. It was found that seven compound peak areas were reduced in the experimental group compared to the control group. These results indicate that these seven compounds (quercetin−3−*O*−vicianoside, quercetin 3−*O*−neohesperidoside, rutin, hyperoside, quercetin−3−*O*−glucuronide, luteolin−7−*O*−neohesperidoside, kaempferol 3−glucuronide) can bind with alpha-glucosidase, and that they may be potential AGIs. Furthermore, the binding degree of the seven potential AGIs was calculated according to Equation (1) and the results are listed in Table 2. All seven components had a good binding degree (>68%), and three of them (hyperoside, quercetin−3−*O*−glucuronide, kaempferol 3−glucuronide) exhibited an excellent alpha−glucosidase binding degree (>90%) [22].

(1) $$\mathrm{binding}\;\mathrm{degree}=\frac{P1-P2}{P2}\times 100\%\;$$

where *P*1 and *P*2 were the peak areas of components in control group (interacting with inactive alpha-glucosidase) and experimental group (interacting with active alpha-glucosidase) in HPLC chromatograms.

Ultrafiltration combined with HPLC, a rapid and solvent-saving technique, was successfully applied in screening bioactive compounds from a complex mixture [6,7,8]. However, the reported ultrafiltration combined with HPLC method using the traditional sample preparation process (ultrasonic, reflux extraction, etc.) is difficult for the simultaneous extraction of different polar components from a complex mixture. In the current method, the multi-step MSPD extraction was selected to extract different polar components from PVR by three different solvents. Seven potential active components were found by multi-step MSPD, ultrafiltration and HPLC. Compared with reported methods, the developed method was a more comprehensive method for screening active components from herbal medicines.

### 2.6. Molecular Docking Studies

**Figure 5:**
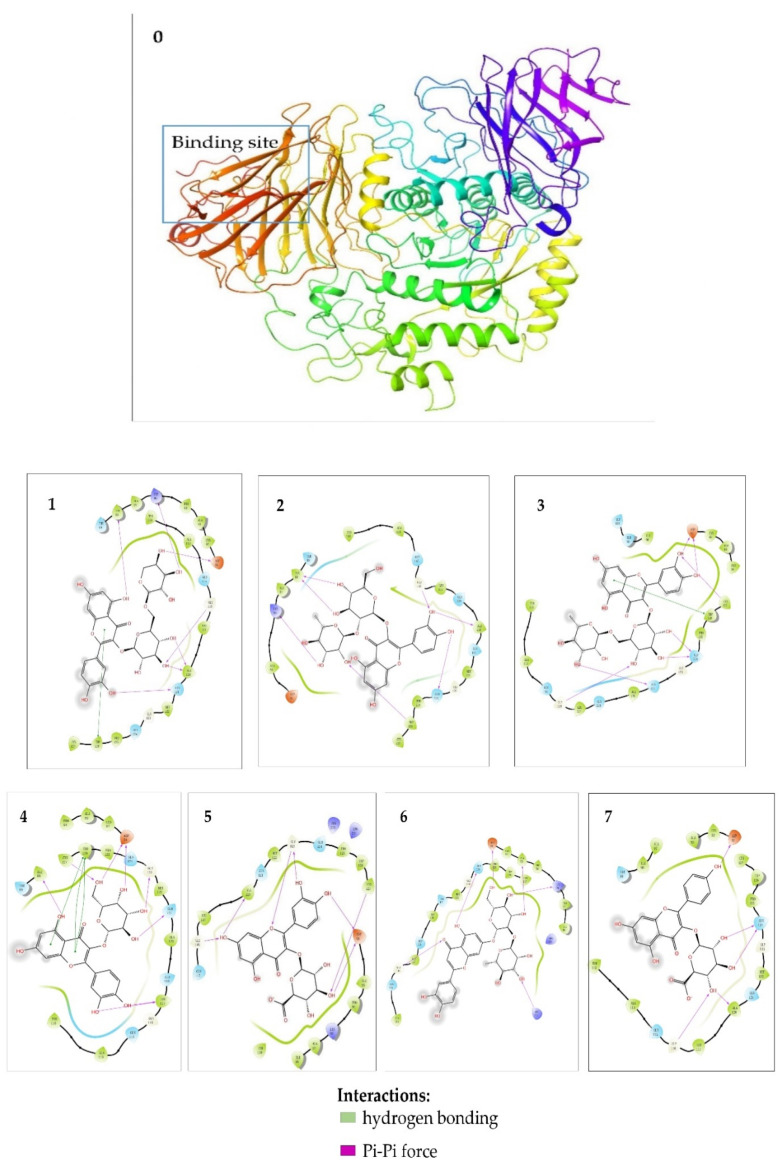
Molecular docking analysis of seven compounds with alpha-glucosidase. Alpha−glucosidase (**0**), quercetin−3−*O*−vicianoside (**1**), quercetin 3−*O*−neohesperidoside (**2**), rutin (**3**), hyperoside (**4**), quercetin 3−*O*−glucuronide (**5**), luteolin−7−*O*−neohesperidoside (**6**), kaempferol 3−glucuronide (**7**).

Molecular docking analysis is a powerful tool for validating the binding sites and binding energy between bioactive components and enzymes. The seven potential AGIs (quercetin−3−*O*−vicianoside, quercetin 3−*O*−neohesperidoside, rutin, hyperoside, quercetin 3−*O*−glucuronide, luteolin−7−*O*−neohesperidoside, and kaempferol 3−glucuronide) were docked with alpha−glucosidase. The binding energy and the hydrogen bonding of the seven components are listed in Table 3. In previous literatures, compounds with binding energy below −5.0 Kcal/mol could be considered as bioactive compounds [23]. The binding energies of these seven components were all lower than −7.0 Kcal/mol. Therefore, these seven components were considered as AGIs. Meanwhile, the results demonstrated that these seven AGIs acted on the active center of alpha-glucosidase via two similar interactions (van der Waals force and Pi-Pi force) (Figure 5). For example, quercetin−3−*O*−vicianoside could insert into the alpha-glucosidase binding pocket based on interactions with residues of Ile98, Lys96, Asp91, Gly116, Ala120, Gln121 via hydrogen bonding and the interactions with residues of Trp126 via Pi-Pi force. Overall, these compounds could bind into the active center of alpha-glucosidase, and exhibit favorable inhibitory effects on the alpha-glucosidase. They were proved as AGIs by molecular docking analysis.

## 3. Materials and Methods

### 3.1. Chemicals and Materials

Rutin, hyperoside, and quercetin−3−*O*−glucuronide were purchased from National Institutes for Food and Drug Control (National Institutes for Food and Drug Control, Beijing, China), Shanghai Standard Technology Co., Ltd. (Standard Technology Corp., Shanghai, China), and Ronghe Pharmaceutical Technology Development Co., Ltd. (Ronghe Pharmaceutical Technology Development Corp., Shanghai, China), respectively. Alpha-glucosidase powder (750 UN) was purchased from Sigma-Aldrich Trading Co., Ltd. (Sigma-Aldrich Trading Corp., Shanghai, China). ρ-nitrophenyl α−d−glucopyranoside (ρNPG) was bought from Yuanye Biotechnology Co., Ltd. (Yuanye Biotechnology Corp., Shanghai, China). Centrifugal ultrafiltration filters (Amicon Ultra−0.5) were provided from Merck Millipore Ltd. (Merck Millipore Corp., Darmstadt, Germany). HPLC-grade acetonitrile and acetic acid were bought from Energy Chemistry Co., Ltd. (Energy Chemistry Corp., Shanghai, China) and Xilong Scientific Co., Ltd. (Xilong Scientific Corp., Shantou, China). ODS−AQ−HG (12 nm S–50 µm, AQG12S50) was purchased from YCM Co., Ltd. (YCM Corp., Kyoto, Japan). Purified water for HPLC-MS was prepared by Milli-Q purification system (Millipore Corp., Billerica, MA, USA). All other chemicals and solvents were of analytical grade.

Polygoni Vivipari Rhizoma (PVR) was collected from Yunnan Province, and authenticated as the dried root of *Polygonum viviparum* by Dr. Zhengming Qian. Voucher specimens were deposited at Bao’an Authentic TCM Therapy Hospital, Shenzhen, China.

### 3.2. Sample Preparation

#### 3.2.1. Preparation of Reference Compound Solutions and Blank Solution

The stock solutions of three reference substances (rutin, hyperoside, and quercetin−3−*O*−glucuronide) were prepared in 3% methanol. Mixed reference compound solutions were prepared by mixing them and diluting to the intended concentration with 3% methanol. The blank solution was 3% methanol solution. All the solutions were stored at 4 °C.

#### 3.2.2. Preparation of the Sample by Multi-Step MSPD

**Figure 6:**
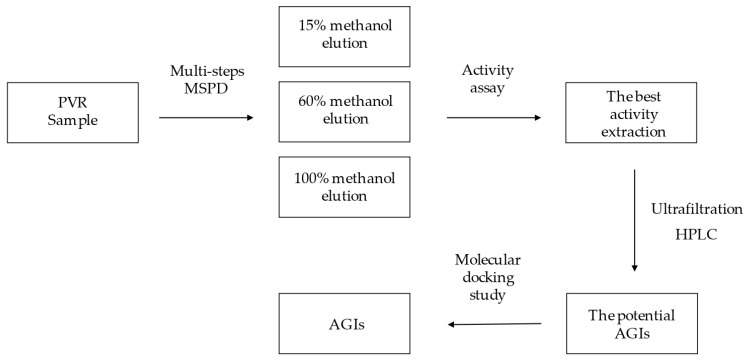
Schematic diagram of multi-step MSPD, ultrafiltration and HPLC.

As shown in Figure 6, the sample was extracted by multi-Step MSPD. The 50 mL MSPD tube (Agilent Technologies, Santa Clara, CA, USA) with a sieve plate at the bottom was filled with 3.0 g ODS. The MSPD tube was eluted with 15% methanol solution before extraction. The PVR sample powders (2.0 g) were sieved and thoroughly dispersed with diatomaceous earth (4.0 g). A 1.0 g mixture was accurately weighed and loaded into the MSPD tube. The sample mixtures were eluted with 40 mL of 15%, 60%, and 100% methanol solution, respectively. The different polar sample elutions were collected, concentrated, lyophilized and stored at 4 °C for further experiments.

### 3.3. Alpha-Glucosidase Inhibitory Assay

The alpha-glucosidase inhibitory assay was carried out according to the reported method with slight modifications [6]. In brief, the lyophilized PVR extractions and alpha-glucosidase were dissolved in phosphate buffer (0.1 mol/L, pH 6.86) and filtered through a 0.22 µm membrane. The PVR solutions (0.78, 1.56, and 3.12 µg/mL, 40 µL) and alpha-glucosidase solution (0.15 U/mL, 40 µL) were mixed and pre-incubated at 37 °C for 15 min. Then, ρNPG (0.5 mg/mL, 80 µL) was added to initiate the reaction. After incubation at 37 °C for 30 min, sodium carbonate (1.0 mol/L, 40 µL) was added to stop this reaction. The solutions were tested in triplicate with a micro-plate reader at 405 nm. The control was tested with the same reaction system, but the PVR solution was replaced by phosphate buffer. The percent of inhibition of alpha-glucosidase activity was calculated as follows [24]:

(2) $$Inhibition\;\left(\%\right)=\frac{A1-A2}{A1}\times 100\%$$

where *A*1 and *A*2 are the absorbance of control and PVR solution.

### 3.4. HPLC-MS Analysis

An Agilent 1260 Series HPLC system (Agilent Technologies, Santa Clara, CA, USA), consisted of an automatic sampler, a QuatPump, a column oven, and a diode array detector (DAD), was used for the analysis. The sample separation was achieved on an Agilent Poroshell 120 SB-Aq column (150 × 3.0 mm, 2.7 µm) at a column temperature of 25 °C. The mobile phase was 0.2% acetic acid (A) and acetonitrile (B) with a flow rate of 0.4 mL/min and used gradient elution: 0–45 min, 11–16% B; 45–47 min, 16–25% B; 47–48 min, 25–65% B. The detection wavelength was set at 360 nm and the injection volume was 5.0 µL.

An Agilent 6530 Series quadrupole time-of-light tandem mass spectrometry (Q-TOF-MS, Agilent Technologies, Santa Clara, CA, USA) was used to identify the compounds in samples. The mass spectrometry parameters were set as follows: electrospray ionization source; negative ion mode; scanning range 50–1200 *m/z*; drying gas (N_2_) flow rate, 11.0 L/min; drying gas temperature, 350 °C; nebulizer pressure, 35 psi; capillary voltage, 4000 V; fragment voltage, 120 V.

### 3.5. Screening Potential AGIs by Ultrafiltration and HPLC Analysis

The screening experiment was carried out by ultrafiltration and HPLC method. In the experimental group, the PVR extraction and alpha-glucosidase were dissolved in phosphate buffer (0.1 mol/L, pH 6.86) and filtered through a 0.22 µm membrane. Then, the PVR solution (2.0 mg/mL, 200 µL) and alpha-glucosidase solution (6.0 U/mL, 100 µL) were mixed and incubated at 37 °C for 30 min. The mixtures were ultrafiltered via a 10 KDa molecular weight cutoff centrifugal ultrafiltration filter at 10,000× *g* rpm for 45 min to separate unbound compounds from alpha-glucosidase-ligand complexes. The ultrafiltration filter was washed three times with phosphate buffer (0.1 mol/L, pH 6.86) at another centrifugal condition (10,000 rpm, 45 min) to completely remove unbound components.

Additionally, inactive alpha-glucosidase (boiled in water for 15 min) was used to replace active alpha-glucosidase for the control group, with the same operation. Finally, the unbound component solution of the control group and experimental group were analyzed by HPLC. The binding degree of each component was calculated as Equation (1): where P1 and P2 were the peak areas of components in control group (interacting with inactive alpha-glucosidase) and experimental group (interacting with active alpha-glucosidase) in HPLC chromatograms.

### 3.6. Molecular Docking Studies

Schrodinger software (Maestro 11.8) was used for in silico molecular docking studies to validate the binding potency of components to alpha-glucosidase. In this process, the structure information of the 7 components (quercetin−3−*O*−vicianoside, quercetin 3−*O*−neohesperidoside, rutin, hyperoside, quercetin 3−*O*−glucuronide, luteolin−7−*O*−neohesperidoside, and kaempferol 3−glucuronide) were obtained from the PubChem platform, and the crystal structure of alpha-glucosidase (PDB ID = 5NN8) was downloaded from the RCSB Protein Data Bank. Then, the alpha-glucosidase bloat and threshold parameters, which determine the volume and extent of the alpha-glucosidase, were specified as default values of 0 and 0.50 Å, respectively. All ligand and unnecessary water were removed, and hydrogen atoms were added. The 3D chemical structures of investigated compounds were drawn and output with minimized energy. At last, the interaction figures were generated and the results of docking were recorded with binding energies and bonding residues.

## 4. Conclusions

In the present study, a novel method combining multi-step MSPD, ultrafiltration and HPLC analysis for screening of AGIs from PVR was established. Seven bioactive components were found and validated via molecular docking studies. The developed method could be a good approach for rapid and overall screening of enzyme inhibitors from herbal medicines. The seven AGIs that were identified could be used as markers for the quality evaluation of PVR, or as bioactive components for the development of related hypoglycemic products.

## Figures and Tables

**Table 1:** The MS data of compounds.

Peak No.	t_R_ (min)	[M-H]^−^ (m/z)	Fragmentation	Molecular Formula	Identification
1	21.008	595.1353	300.0291, 271.0263, 255.0315, 151.0047	C_26_H_28_O_16_	Quercetin−3−*O*−vicianoside
2	26.434	609.1494	300.0291, 271.0255	C_27_H_30_O_16_	Quercetin 3−*O*−neohesperidoside
3	28.487	609.1439	300.0291, 271.0255	C_27_H_30_O_16_	Rutin
4	29.233	463.0860	300.0245, 271.0221, 151.0007	C_21_H_20_O_12_	Hyperoside
5	31.379	477.0654	384.9314, 301.0330, 255.0279, 151.0014	C_21_H_18_O_13_	Quercetin−3−*O*−glucuronide
6	33.952	593.1520	284.0324, 255.0301, 227.0344	C_27_H_30_O_15_	Luteolin−7−*O*−neohesperidoside
7	42.750	461.0755	285.0414, 257.0462, 229.0521	C_21_H_18_O_12_	Kaempferol 3−glucuronide

**Table 2:** The binding degree of compounds with alpha-glycosidase.

Peak No.	Compounds	Binding Degree (%)	Peak No.	Compounds	Binding Degree (%)
1	Quercetin−3−*O*−vicianoside	68.70	5	Quercetin−3−*O*−glucuronide	96.29
2	Quercetin 3−*O*−neohesperidoside	83.15	6	Luteolin−7−*O*−neohesperidoside	71.35
3	Rutin	78.97	7	Kaempferol 3−glucuronide	91.63
4	Hyperoside	100.00			

**Table 3:** The molecular docking analysis of compounds.

Compounds	Binding Energy (Kcal/mol)	Amino Acid Residues	Hydrogen Bonds
Quercetin−3−*O*−vicianoside	−7.204	Asp91, Lys96, Ile98, Gly116, Ala120, Gln121, Trp126	Asp91, Lys96, Ile98, Gly116, Ala120, Gln121
Quercetin 3−*O*−neohesperidoside	−7.032	Lys96, Ile98, Gly116, Ala120, Gln124, Trp126	Lys96, Ile98, Gly116, Ala120, Gln124, Trp126
Rutin	−7.842	Asp91, Gly116, Gln121, Gln124, Trp126, Cys27	Asp91, Gly116, Gln121, Gln124, Cys127
Hyperoside	−7.922	Asp91, Ile98, Ieu117, Gln121, Gly123, Trp126, Cys127	Asp91, Ile98, Ieu117, Gln121, Gly123, Cys127
Quercetin 3−*O*−glucuronide	−7.718	Asp91, Gly116, Ala120, Gly123, Cys127	Asp91, Gly116, Ala120, Gly123, Cys127
Luteolin−7−*O*−neohesperidoside	−7.948	Asp91, Ala93, Lys96, Gly116, Gln124, Arg275	Asp91, Ala93, Lys96, Gly116, Gln124, Arg275
Kaempferol 3−glucuronide	−7.250	Asp91, Gly116, Ala120, Gln124	Asp91, Gly116, Ala120, Gln124

## Data Availability

The data presented in this study are contained within the article.

## Sample Availability

Samples of the compounds **3**–**5** are available from the authors.
